# Pretreatment with different doses of oliceridine attenuates etomidate-induced myoclonus during painless gastroscopy: a randomized controlled trial

**DOI:** 10.3389/fphar.2026.1664835

**Published:** 2026-05-14

**Authors:** Yingchuan Lin, Yun Dai, Weifeng Huang, Huaijian Zhang, Bin Yang

**Affiliations:** 1 Department of Anesthesiology, The First Affiliated Hospital of Xiamen University, School of Medicine, Xiamen University, Xiamen, Fujian, China; 2 Department of Endoscopy, The First Affiliated Hospital of Xiamen University, School of Medicine, Xiamen University, Xiamen, Fujian, China; 3 Department of Gastroenterology, The First Affiliated Hospital of Xiamen University, School of Medicine, Xiamen University, Xiamen, Fujian, China

**Keywords:** etomidate, gastroscopy, myoclonus, oliceridine, sedation

## Abstract

**Background:**

Etomidate is commonly used for sedation during gastroscopy but is associated with a high incidence of myoclonus. This study evaluated the effectiveness of oliceridine pretreatment in reducing etomidate-induced myoclonus.

**Methods:**

In this prospective, double-blind, randomized controlled trial, 150 patients undergoing painless gastroscopy were assigned to receive oliceridine at 0.01 mg/kg (Group A_1_), 0.02 mg/kg (Group A_2_), or normal saline (Group S) prior to etomidate administration. Remifentanil (0.2 μg/kg) and etomidate (0.3 mg/kg) were then administered. The incidence and severity of myoclonus were assessed within 60 s post-etomidate injection. Hemodynamic parameters, adverse effects, and satisfaction scores were also evaluated.

**Results:**

The incidence of myoclonus was significantly lower in Groups A_1_ (28%) and A_2_ (16%) compared to Group S (58%) (*p* < 0.001). Severe myoclonus occurred less frequently in the oliceridine groups. Heart rate (HR) at T_1_ and T_2_ was significantly lower in Groups A_1_ and A_2_ than in Group S (*p* < 0.05). No significant intergroup differences were observed in mean arterial pressure (MAP) or pulse oxygen saturation (SpO_2_). Injection pain, hiccups, and cough were less frequent in the oliceridine groups, although not statistically significant.

**Conclusion:**

Pretreatment with oliceridine effectively reduces the incidence and severity of etomidate-induced myoclonus during painless gastroscopy without compromising hemodynamic stability.

**Clinical Trial Registration:**

http://www.chictr.org.cn, identifier ChiCTR2500103005.

## Introduction

As non-operating room anesthesia (NORA) becomes increasingly prevalent, optimizing sedation protocols for gastrointestinal endoscopy has become a clinical priority. Etomidate, a frequently used sedative for endoscopic procedures, offers advantages such as rapid onset, brief recovery period, and minimal cardiopulmonary suppression (Song et al., 2015; Tang et al., 2024). Despite these benefits, its use is often limited by adverse events, most notably myoclonus, which occurs in 50%–80% of patients (Hong and Park, 2023; Lang et al., 2019; Gao et al., 2024). Etomidate-induced myoclonus may cause muscle damage, elevate the risk of aspiration, and complicate procedural conditions (Hao et al., 2020; Zhang et al., 2022).

Existing pharmacological interventions for mitigating etomidate-induced myoclonus show inconsistent efficacy, with opioids showing the most promising results (Zhang et al., 2022). Opioids can reduce myoclonus by activating μ-opioid receptors and modulating GABAergic signaling in the basal ganglia. Oliceridine, a novel G protein-biased μ-opioid receptor agonist, has shown promise in providing potent analgesia while minimizing typical opioid-associated adverse events such as respiratory depression (Moss et al., 2023; Markham, 2020).

However, its role in preventing etomidate-induced myoclonus has not been systematically investigated. This study was designed to evaluate whether oliceridine pretreatment decreases the incidence and severity of etomidate-induced myoclonus, while maintaining hemodynamic stability and patient safety.

## Data and methods

### General data

This investigation employed a prospective, randomized, double-blind design to evaluate pretreatment efficacy. The trial was conducted in accordance with the Consolidated Standards of Reporting Trials (CONSORT) guidelines. This research was authorized by the Ethics Committee of the First Affiliated Hospital of Xiamen University (2025 [086]) and registered at http://www.chictr.org.cn (ChiCTR2500103005). Patients who underwent painless gastroscopy at the Endoscopy Center of the First Affiliated Hospital of Xiamen University from May 2025 to July 2025 were included in this study. All patients or their family members signed informed consent forms. The study population consisted of patients aged 19 to 65 years with American Society of Anesthesiologists (ASA) physical status classification I or II.

**FIGURE 1:**
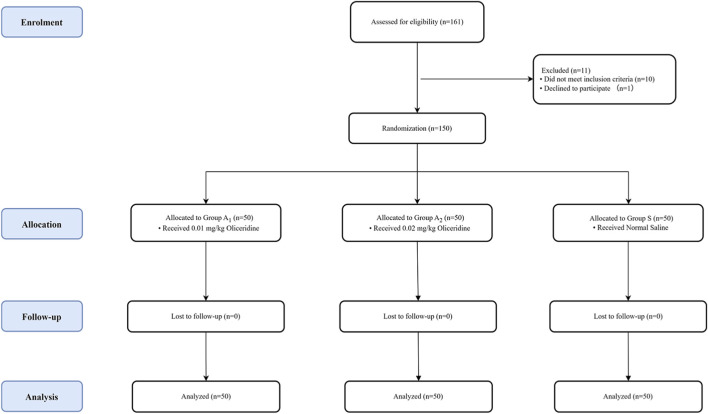
Study flow diagram.

The exclusion criteria were as follows: (1) neuromuscular conduction-disorder diseases; (2) allergy to study medications (etomidate, oliceridine, or remifentanil); (3) diagnosis of epilepsy; (4) recent upper respiratory tract infection (within 14 days); (5) severe dysfunction of vital organs; (6) uncontrolled hypertension (blood pressure > 180/110 mmHg); (7) predicted difficult airway management; (8) obesity (BMI>30 kg/m^2^); (9) chronic use of psychotropic medications or cognitive impairment; (10) inability to cooperate. The research flow chart is shown in Figure 1.

### Methods

All patients in the three groups were required to maintain an 8-h fasting period and 2-h fluid restriction prior to the procedure. Intravenous access was established using a 22-gauge cannula inserted into the dorsal hand vein. No additional drugs were administered before oliceridine administration. Fifteen minutes before the endoscopic procedure, patients received dyclonine hydrochloride mucilage for oropharyngeal anesthesia. All eligible patients were randomly assigned in a 1:1:1 ratio to Group A_1_ (0.01 mg/kg oliceridine), Group A_2_ (0.02 mg/kg oliceridine), or Group S (0 mg/kg oliceridine, equivalent volume of normal saline) (n = 50 per group) using a random number table administered by nursing staff. Following patient admission, vital parameters including MAP, HR, and SpO_2_ were continuously monitored, with MAP being automatically computed by the monitoring device. The patients were instructed to lie in the left lateral position and were given nasal catheter oxygen inhalation at a rate of 3 L/min. The grouping information was sealed in an envelope, and a nurse who was not involved in the study prepared the opioid drugs and normal saline. This ensured that the patients and anesthesiologists were all unaware of the grouping.

Group A_1_ received an intravenous administration of 0.01 mg/kg oliceridine, while Group A_2_ was administered 0.02 mg/kg oliceridine intravenously. Group S served as the control group, receiving an equivalent volume of normal saline intravenously. Following a 2-min interval, all patients in the three groups received intravenous remifentanil (0.2 μg/kg) administered over a minimum of 60 s, followed by intravenous etomidate (0.3 mg/kg) delivered over 20 s. Patients’ myoclonus severity was assessed by a second anesthesiologist within 60 s following etomidate administration, using a standardized rating scale. Gastroscopy commenced following achievement of target sedation (eyelash reflex disappearance). Throughout the endoscopic examination, supplemental sedation with etomidate (2–4 mg intravenous boluses) was administered in response to patient movement, coughing, or tachycardia, with repeated dosing as clinically required to maintain optimal procedural conditions. When SpO_2_ decreased below 90%, airway patency was established using jaw-thrust technique, followed by positive pressure ventilation with 100% oxygen via a resuscitation bag if required. Symptomatic bradycardia (HR < 45 beats/min) was treated with intravenous atropine (initial dose 0.5 mg) or isoproterenol infusion (1–2 μg/min), titrated to clinical response. The anesthesia and endoscopy procedures were respectively completed by anesthesiologists and endoscopists with more than 5 years of working experience.

### Observation indicators

#### Primary indicators

The degree of myoclonus after etomidate administration was observed and scored. Using the grading where 0 = no myoclonus, 1 = mild myoclonus (only mild fasciculation involving the face and/or distal upper and/or lower extremities), 2 = moderate myoclonus (marked movements of the face and/or limbs) and 3 = severe myoclonus (involving the limbs and trunk) (Rautela et al., 2023).

### Secondary indicators

MAP, HR, and SpO_2_ were monitored and recorded at four key time points: baseline (T_0_, pre-pretreatment), 2 min post-oliceridine/saline administration (T_1_), immediately after etomidate injection (T_2_), and at the time of endoscopic esophageal insertion (T_3_). After etomidate administration, within the first 10 s, patients were asked to rate the pain intensity associated with the injection using an 11-point Pain Intensity Numerical Rating Scale (PI-NRS), where 0 indicated no pain and 10 represented the worst pain possible (Kang et al., 2010). Additionally, the occurrence of hiccup and cough reflexes was recorded during the period from etomidate injection to the completion of the gastroscopy. A systematic telephone follow-up was performed 24 h postoperatively to evaluate the incidence of nausea and vomiting. Patient satisfaction with the gastroscopy experience was assessed in the post anesthesia care unit (PACU) 30 min following emergence, utilizing a 4-point scale (4 = no discomfort; 3 = mild discomfort; 2 = extreme discomfort; 1 = unacceptable discomfort). Endoscopist satisfaction was evaluated upon procedure completion using a separate 4-point scale (4 = very good; 3 = good; 2 = fair; 1 = poor) (Yang et al., 2022).

### Statistical analysis

The sample size calculation was performed with PASS software (version 15.0; NCSS, Kaysville, UT). According to existing literature, the incidence of myoclonus induced by etomidate was as high as 72% (Shan et al., 2023). Preliminary experimental findings indicated that pretreatment with 0.2 μg/kg remifentanil reduced this incidence to 53%, which was consequently adopted as the expected baseline rate for the control group. Power analysis demonstrated that 47 participants per group would provide 90% power to detect a 30% reduction in myoclonus incidence at a one-sided α = 0.025, accounting for a 5% attrition rate. To ensure adequate statistical power following potential participant dropouts, the sample size was increased to 50 participants per group, yielding a total sample size of 150.

Continuous variables were assessed for normality using the Shapiro-Wilk test. Normally distributed variables were presented as mean ± standard deviation (SD), while non-normally distributed variables were expressed as median with interquartile range (IQR; 25th, 75th percentiles). Categorical variables were reported as frequencies and percentages (%). Continuous variables were analyzed using t-tests/Wilcoxon tests (two groups) or ANOVA/Kruskal-Wallis tests (multiple groups). Categorical variables were compared via chi-square or Fisher’s exact tests, as appropriate. Post-hoc pairwise comparisons were performed with Bonferroni correction for multiple testing. All analyses were conducted using SPSS Statistics 26.0 (IBM Corp.). A two-tailed p-value <0.05 was considered statistically significant.

## Results

### Baseline characteristics

In the present study, 161 patients were screened for eligibility, of whom 150 met the inclusion criteria and were subsequently enrolled and randomized into three groups. No significant differences were observed in baseline characteristics, including gender distribution, age, height, weight, BMI, and ASA classification among the three groups (Table 1). In terms of endoscopist satisfaction, both Group A_1_ and Group A_2_ exhibited relatively higher levels compared to Group S. However, this difference was not statistically significant. Similarly, no significant differences were observed in patient satisfaction across the three groups (Table 1).

**TABLE 1:** Demographic characteristics and satisfaction scores across the three groups.

Variable	Group A_1_ (n = 50)	Group A_2_ (n = 50)	Group S (n = 50)	P value
Gender (female/male)	29/21	30/20	28/22	0.46
Age (year)	43.86 ± 10.99	43.92 ± 9.67	42.36 ± 10.89	0.73
Height (cm)	1.64 ± 0.09	1.63 ± 0.09	1.66 ± 0.08	0.82
Weight (kg)	59.65 ± 10.02	62.65 ± 12.03	61.60 ± 11.86	0.65
BMI (kg/m^2^)	22.14 ± 2.83	23.18 ± 2.69	22.21 ± 3.03	0.79
ASA (I/II)	26/24	29/21	28/22	0.22
Patient satisfaction	4 [3–4]	4 [3–4]	4 [3–4]	0.99
Endoscopist satisfaction	4 [3–4]	4 [3–4]	3 [2–4]	0.07

Data are mean (standard deviation), median [range] or n. Group A_1_ = 0.01 mg/kg oliceridine; Group A_2_ = 0.02 mg/kg oliceridine; Group S = 0 mg/kg oliceridine. Student’s t-test or Mann-Whitney U was utilized to assess differences.

### Primary outcome

The incidence of myoclonus in Group A_1_ and Group A_2_ was significantly lower than that in Group S (Group A_1_: 28%, Group A_2_: 16%, Group S: 58%, *p* < 0.001). Furthermore, a detailed severity analysis of myoclonus demonstrated that both Group A_1_ and Group A_2_ exhibited significantly lower rates of severe myoclonus compared to Group S (Table 2). However, no statistically significant intergroup differences were observed in the incidence of mild or moderate etomidate-induced myoclonus. The incidence of myoclonus was numerically higher in female patients (37.9%) compared to male patients (26.9%), with this 11.0 percentage-point difference not reaching statistical significance *p* = 0.160 (Table 3).

**TABLE 2:** Incidence of etomidate-induced myoclonus.

Variable	Group A_1_ (n = 50)	Group A_2_ (n = 50)	Group S (n = 50)	$\chi^{2}$	P value
Incidence [no. (%)]	14 (28%)^*^	8 (16%)^#^	29 (58%)	22.1	*p* < 0.001
The severity of myoclonus [no. (%)]	​	​	​	44.5	*p* < 0.001
None	36 (72%)^*^	42 (84%)^#^	21 (42%)	​	​
Mild	10 (20%)	6 (12%)	8 (16%)	​	​
Moderate	3 (6%)	2 (4%)	11 (22%)	​	​
Severe	1 (2%)^*^	0 (0)^#^	10 (20%)	​	​

Data are n (%) or n. Compared with Group S.

^*^ *p* < 0.05; compared with Group S.

^*#*^ *p* < 0.05. Group A_1_ = 0.01 mg/kg oliceridine; Group A_2_ = 0.02 mg/kg oliceridine; Group S = 0 mg/kg oliceridine. Chi-square test or Fisher’s exact test was utilized to assess differences.

**TABLE 3:** Comparative analysis of etomidate-induced myoclonus incidence by gender.

Variable	Male (n = 63)	Female (n = 87)	$\chi^{2}$	P value
Incidence [no. (%)]	17 (26.9%)	33 (37.9%)	1.97	0.160
The severity of myoclonus [no. (%)]	​	​	4.91	0.178
None	46 (73.0%)	54 (62.0%)	​	​
Mild	8 (12.6%)	15 (17.2%)	​	​
Moderate	6 (9.5%)	10 (11.4%)	​	​
Severe	3 (4.7%)	8 (9.1%)	​	​

Data are n (%) or n. Chi-square test or Fisher’s exact test was utilized to assess differences.

### Secondary outcome

The incidence of injection pain was significantly lower in Group A_1_ (1/50, 2%) and Group A_2_ (2/50, 4%) compared to Group S (6/50, 12%), with the difference approaching statistical significance (*p* = 0.057). Additionally, the incidence of hiccup among the three groups demonstrated no significant statistical differences. In terms of cough, 2 cases were reported in Group S and 1 case in Group A_2_. No nausea or vomiting were observed in three groups (Table 4).

**TABLE 4:** Incidence of adverse-effects.

Variable	Group A_1_ n (%)	Group A_2_ n (%)	Group S n (%)	$\chi^{2}$	P value
Injection site pain	1 (2)	2 (4)	6 (12)	5.74	0.057
Hiccup	11 (22)	6 (12)	13 (26)	4.63	0.099
Cough	0	1 (2)	2 (4)	-	0.367
Nausea-vomiting	0	0	0	-	1.000

Data are n (%) or n. Group A_1_ = 0.01 mg/kg oliceridine; Group A_2_ = 0.02 mg/kg oliceridine; Group S = 0 mg/kg oliceridine. Chi-square test or Fisher’s exact test was utilized to assess differences.

**FIGURE 2:**
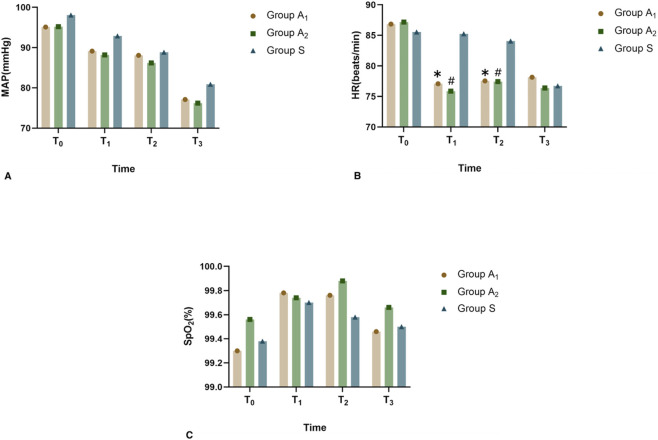
Vital signs change after pretreatment. **(A)** MAP (mmHg); **(B)** HR (beats/min); **(C)** SpO_2_; Group A_1_ = 0.01 mg/kg oliceridine; Group A_2_ = 0.02 mg/kg oliceridine; Group S = 0 mg/kg oliceridine. Compared with Group S, ^*^ *p* < 0.05; compared with Group S, ^*#*^ *p* < 0.05. Abbreviations: MAP, mean arterial pressure; HR, heart rate; SpO_2_, saturation of pulse oximetry; T_0_, time before administration of oliceridine or normal saline injection; T_1_, 2 min after administration of oliceridine or normal saline injection; T_2_, etomidate injection; T_3_, at the time of endoscopic esophageal insertion.

Compared to Group S, both Group A_1_ and Group A_2_ showed significantly lower HR at time points T_1_ and T_2_ (*p* < 0.05). However, there were no significant differences in MAP and SpO_2_ between the groups (Figure 2).

## Discussion

This study demonstrated that pretreatment with oliceridine (0.01–0.02 mg/kg) significantly reduced the incidence and severity of etomidate-induced myoclonus without exacerbating typical etomidate-related adverse effects. While the 0.02 mg/kg group showed numerically lower myoclonus rates compared to the 0.01 mg/kg group (16% vs. 28%), this difference lacked statistical significance (*p* > 0.05), suggesting limited dose-dependent efficacy within this dosage range. These findings aligned with previous studies that showed low-dose opioid pretreatment mitigated etomidate-induced myoclonus (Zhang et al., 2022). Notably, a gender disparity in myoclonus incidence was observed (37.9% in females vs. 26.9% in male), which corroborated earlier findings reported by Stockham et al. (1988). This difference was potentially attributable to hormonal or neuroanatomical modulation of myoclonic responses. A standardized low-dose remifentanil regimen was administered to all three groups, consistent with established evidence demonstrating its effectiveness in attenuating etomidate-induced myoclonus (Kelsaka et al., 2006; Lee et al., 2009; Ri et al., 2011). The inclusion of remifentanil was particularly crucial for the control group, as its omission would have resulted in substantially higher myoclonus incidence rates.

Existing literature supports the efficacy of opioid pretreatment in mitigating etomidate-induced myoclonus, though each agent presents clinical limitations. Stockham et al. (1988) observed dose-dependent myoclonus reduction with fentanyl, yet higher doses (500 μg) invariably caused apnoea. Sufentanil (0.3 μg/kg) eliminated myoclonus but was associated with significant respiratory depression (30% incidence) (Hueter et al., 2003). Butorphanol (0.015 mg/kg) demonstrated suboptimal efficacy (70% myoclonus incidence) with delayed onset (Rautela et al., 2023). Remifentanil, despite its advantageous pharmacokinetic profile featuring rapid onset, and short duration, carries intrinsic risks of chest wall rigidity and apnoea (Ri et al., 2011).

Non-opioid agents also have some effect in preventing etomidate-induced myoclonus. Gao et al. reported that esketamine showed limited efficacy against mild myoclonus while potentially increasing intracranial pressure, intraocular pressure, and hallucination risk (Gao et al., 2024; Wang et al., 2024). Pretreatment with etomidate (0.05 mg/kg) proved ineffective in reducing myoclonus incidence (Schwarzkopf et al., 2003). Additionally, Miao et al. observed that dexmedetomidine (0.5 μg/kg) reduced myoclonus by 38%, but with prolonged onset time (Miao et al., 2019). Midazolam, with an 84% failure rate, demonstrated substantial limitations in alleviating myoclonus (Alipour et al., 2016).

The pathophysiology of etomidate-induced myoclonus likely involves cortical disinhibition, where early cortical suppression unmasks subcortical hyperexcitability in motor pathways (Reddy et al., 1993; Doenicke et al., 1999). Opioid-mediated inhibition of myoclonus likely occurs through μ-opioid receptor (MOR)-dependent modulation of GABAergic signaling in basal ganglia circuits (Zhang et al., 2022; Doenicke et al., 1999). Oliceridine’s unique pharmacological profile as a G protein-biased MOR agonist with limited β-arrestin recruitment offers dual advantages: potent analgesia through Gq signaling coupled with reduced respiratory depression due to minimal β-arrestin pathway activation (Ni et al., 2024; Spahn and Stein, 2017). This mechanistic selectivity aligns with the observed stable SpO_2_ levels across groups. The combination of oliceridine’s rapid onset (peak analgesia: 5 min) and short elimination half-life (1.3–3 h) (Markham, 2020) synergizes well with etomidate’s pharmacokinetic profile, making this regimen particularly suitable for ambulatory endoscopic procedures.

Currently, research on the dosage of oliceridine in intraoperative anesthetic management remains limited. Most studies have adopted a low dose of 0.02 mg/kg (Cao et al., 2025), which has been verified to be associated with a low incidence of adverse reactions. Accordingly, the present study employed oliceridine at doses of 0.01 mg/kg and 0.02 mg/kg.

Pretreatment with 0.02 mg/kg oliceridine additionally reduced hiccup incidence (potentially through diaphragmatic suppression) and improved endoscopist satisfaction by minimizing patient movement. Notably, the attenuated injection pain in oliceridine groups may reflect the agent’s intrinsic analgesic properties. A lower HR was observed in the oliceridine groups at T_1_ and T_2_ time points, which might be attributed to opioid-mediated sedative effects (Montandon and Horner, 2019). Interestingly, one patient in the Group A_2_ experienced coughing during the procedure, which was subsequently attributed to mechanical stimulation from a glottis-proximal polyp identified during gastroscopy.

Several limitations should be acknowledged in our study. First of all, the lack of continuous respiratory rate monitoring and apnoea detection prevented a more precise evaluation of the respiratory effects associated with different oliceridine doses. Secondly, the study only included the general population and did not conduct research on high-risk groups for nausea and vomiting. This population selection bias may account for the observed absence of nausea and vomiting in our study. Thirdly, adrenal cortical function was not assessed in this study. Although etomidate has an inhibitory effect on adrenal cortical function, some studies have shown that a single intravenous injection of etomidate does not cause long-term adrenal cortical function inhibition (Cotten et al., 2009). Postoperative recovery of adrenal-related hormone levels occurs rapidly, and etomidate administration has not been associated with increased risk of in-hospital cardiovascular, gastrointestinal, or infectious complications, nor with increased overall mortality during hospitalization (Lu et al., 2022). Fourthly, the present investigation was confined to two specific doses of oliceridine for etomidate-induced myoclonus. Further research with the optimal clinical dose needed to study.

## Conclusion

Pretreatment with oliceridine (0.01 mg/kg, 0.02 mg/kg) significantly alleviates etomidate-induced myoclonus during painless gastroscopy. This regimen exhibits no significant effects on vital signs, with a low incidence of adverse reactions and favorable clinical safety. Therefore, oliceridine may serve as a preferred adjuvant strategy for anesthesia induction with etomidate and is worthy of clinical application.

## Data Availability

The original contributions presented in the study are included in the article/supplementary material, further inquiries can be directed to the corresponding author.
